# Key Nutrients for Optimal Blood Glucose Control and Mental Health in Individuals with Diabetes: A Review of the Evidence

**DOI:** 10.3390/nu15183929

**Published:** 2023-09-10

**Authors:** Raedeh Basiri, Blessing Seidu, Lawrence J. Cheskin

**Affiliations:** 1 Department of Nutrition and Food Studies, George Mason University, Fairfax, VA 22030, USA; 2 Institute for Biohealth Innovation, George Mason University, Fairfax, VA 22030, USA; 3 Department of Medicine, Johns Hopkins School of Medicine, Baltimore, MD 21205, USA

**Keywords:** diabetes, mental health, anxiety, depression, supplementation, nutrients, blood glucose, nutrition, omega-3 fatty acids, vitamin D, vitamin E, vitamin B6, vitamin B12, folate, selenium, chromium, iron, magnesium

## Abstract

Diabetes is associated with an increased risk of mental disorders, including depression, anxiety, and cognitive decline. Mental disorders can also contribute to the development of diabetes through various mechanisms including increased stress, poor self-care behaviors, and adverse effects on glucose metabolism. Consequently, individuals suffering from either of these conditions frequently experience comorbidity with the other. Nutrition plays an important role in both diabetes and mental health disorders including depression and anxiety. Deficiencies in specific nutrients such as omega-3 fatty acids, vitamin D, B vitamins, zinc, chromium, magnesium, and selenium have been implicated in the pathogenesis of both diabetes and mental disorders. While the impact of nutrition on the progression and control of diabetes and mental disorders is broadly acknowledged, there is a notable knowledge gap concerning the implications of distinct nutrients in preventing and mitigating symptoms of both conditions when they coexist. The aim of this study was to examine the role of nutrition in improving glucose homeostasis and promoting mental well-being among individuals with diabetes. Further, we evaluated the preventive or delaying effects of key nutrients on the simultaneous manifestation of these conditions when one of them is present. Our findings indicated that the use of personalized dietary interventions and targeted nutrient supplementation can improve metabolic and mental health outcomes in patients with type 2 diabetes.

## 1. Introduction

The prevalence of diabetes has more than doubled in recent years, making it one of the most devastating diseases of the 21st century [1]. In 2021, the estimated global diabetes prevalence stood at 537 million, with projections indicating a rise to 643 million by 2030, and a further increase to 783 million by 2045 [2]. The mortality rate of diabetes is alarmingly high. It has been reported that 1.5 to 5.1 million people per year lost their lives due to diabetes and related complications, placing it as the 8th leading cause of death worldwide [3]. According to the United Kingdom Prospective Diabetes Study (UKPDS) clinical trial, strict glycemic regulation decreases the risk of developing diabetes complications [4] and can significantly improve comorbid conditions, such as mental health disorders [5].

Major depression and anxiety have often been linked to diabetes [6,7]. While some researchers have shown that diabetes is a risk factor for developing depression and anxiety [8], other studies have found that depression and stress are risk factors for type 2 diabetes [9,10]. Depression and anxiety can negatively affect the quality of life of individuals due to the impact of symptoms and adverse effects [11]. Chronic stress can augment the production of inflammatory cytokines directly or through the hypothalamic–pituitary–adrenocortical (HPA) axis, which negatively affects the functioning of pancreatic β-cells, creates insulin resistance, and ultimately can lead to diabetes [12]. Anxiety and depression have also been noted to negatively influence eating patterns and food choices [13]. This behavior may contribute to adiposity and the risk of developing diabetes [11]. Changes in appetite and a lack of interest in physical activity (behavior) can further trigger the development of diabetes [12].

In addition, diabetes self-management, which typically entails the perpetual management of blood glucose levels and sometimes taking insulin injections, may increase emotional burden and mental disturbances [14,15], which can result in depressive and anxiety symptoms. Although the best quality of care among individuals with diabetes typically helps improve diabetes symptoms, other factors heighten the risk of developing depression and anxiety, such as worry about the increase in morbidity and mortality of the condition, developing related complications, and the risk of hypoglycemia [16,17]. The incidence of depression has been noted to be high among people with diabetes compared to people with normal glycemic levels [18]. Depression impacts one in four adults with diabetes, while only 25% to 50% of these individuals are diagnosed and receive treatment [6]. One way to prevent or alleviate symptoms of such conditions is through a well-balanced diet and supplementation with essential nutrients. Making changes to one’s lifestyle, including diet, can significantly reduce the onset of many chronic health conditions and can also help alleviate symptoms if they are already present [13]. This literature review will assess how improved nutrition can prevent or alleviate symptoms of mental disorders and diabetes.

## 2. Materials and Methods

A comprehensive literature review was performed to investigate current evidence of the effects of different nutrients in preventing or treating mental health disorders and/or diabetes. Inclusion criteria included observational studies, randomized control trials (RCTs), systematic reviews, and meta-analyses published between 2008 and 2023. Databases were accessed and searches were performed through the George Mason University Library, PubMed, and Google Scholar. The keywords used in our search strategy were “depression” or “anxiety” or “mental health” or “diabetes mellitus”, and “nutrition”, “macronutrients” or “omega-3 fatty acids” or “vitamin B6” or “folate” or “B12” or “vitamin D” or “vitamin E” or “zinc” or “chromium” or “selenium” or “magnesium” or “iron”.

## 3. Results

Optimizing nutritional status is a key component of diabetes management, mental health, and general well-being [19]. Over the past decade, dietary intake has been considered a modifiable risk factor for mitigating the symptoms of diabetes, depression, and anxiety [20]. However, it’s important to note that more clinical trials need to be conducted to confirm the effects of specific nutrients on diabetes and mental health outcomes when they coexist. It has been shown that individuals with diabetes who follow a specialized diet can maintain the best glycemic control and decrease the chance of diabetes-related complications [21,22,23,24,25,26,27,28]. In addition, the poor nutritional intake of essential nutrients can impact the body’s ability to produce hormones and neurotransmitters and further influence blood glucose levels and mental health [29]. Polyunsaturated fatty acids, notably omega-3, vitamin D, vitamin E, B vitamins, zinc, magnesium, chromium, selenium, and iron are the most important nutrients for improving mental health, blood glucose, and diabetes-related complications [30,31]. Simple sugars and saturated fats, on the other hand, can be hazardous to cerebral function and contribute to diabetes and related complications [32,33]. See Table 1 for various recommended intakes.

### 3.1. Carbohydrates, Fibers, and Fats

Over the years, studies have pointed to reducing or restricting carbohydrate intake, particularly simple carbohydrates, in preventing diabetes and reducing anxiety [34]. Carbohydrates are important macronutrients for the body and brain [35]; however, a high intake of refined carbohydrates is associated with cognitive impairment, emotional stress, and negatively affects brain function and overall health [36]. A limited number of studies have been conducted to evaluate the impact of either low or high carbohydrate consumption, as well as the effects of glycemic index (GI) and glycemic load (GL), on depressive symptoms in individuals with diabetes. In a cross-sectional study by Umegaki et al., a high intake of carbohydrates was associated with depression in women but not men [37]. Similarly in a study by Mwamburi et al., it was found that depressed patients with diabetes consumed a diet with a higher GI than non-depressed individuals [38]. Diets with higher glycemic indices have been linked to a reduced diet quality and diminished intake of essential nutrients for depression, such as magnesium, riboflavin, and dietary folate equivalents [39]. Moreover, diets with elevated glycemic indices are predominantly associated with the decreased consumption of nutrient-rich foods, including vegetables, fruits, and dietary fiber [40]. Therefore, it is generally recommended that diet therapy should limit the quantity of refined carbohydrates and increase diet quality and dietary fiber consumption [41].

Studies suggest that frequent intake of dietary fiber mitigates anxiety [42], slows the absorption of carbohydrates, and enhances insulin sensitivity, thus better controlling blood glucose [43]. Dietary fiber also has the potential to change the gut microbiota [44]. It interacts with the gut microbiota to produce short-chain fatty acids (SCFA), which help improve glucagon-like peptide 1 (GLP-1) and blood glucose levels [45]. The positive outcome of SCFA on blood sugar regulation, body mass index (BMI), resting energy expenditure, and lipolysis has been shown in both animal [46] and human [47] studies. The gut microbiota has also been shown to play a critical role in mental health [48]. Microbiome studies have indicated that SCFA-producing probiotics such as Bifidobacterium longum, Lactobacillus rhamnosus, and Clostridium butyricum strains have a positive effect on various psychiatric disorders, including anxiety and depression [29,49,50]. Overall, study findings point to the need for limiting the intake of simple carbohydrates and a high intake of dietary fiber for improved anxiety and diabetes care.

### 3.2. Polyunsaturated Fatty Acids

Omega-3 fatty acids have shown positive effects on mental health [30]. Additionally, long-chain omega-3 polyunsaturated fatty acids (PUFAs), eicosapentaenoic acid (EPA; 20:5*n* − 3) and docosahexaenoic acid (DHA; 22:6*n* − 3) have favorable relationships with risk factors for diabetes, mental health disorders, inflammation, and adiposity [31]. Moreover, PUFA-derived specialized proresolving lipid mediators play a significant role in both diabetes and depression. In diabetes, these mediators contribute to the resolution of inflammation associated with chronic hyperglycemia and insulin resistance, potentially helping to mitigate the progression of diabetic complications [32]. In depression, these lipid mediators are thought to influence neuroinflammatory processes and synaptic plasticity, suggesting a link between their dysregulation and the development of depressive symptoms. Understanding the intricate interplay of these mediators in both conditions could hold therapeutic promise for managing the inflammatory aspects of diabetes and the neuroinflammatory components of depression [33].

Although EPA and DHA have distinct functions, they perform best when combined. For instance, DHA is known to lower the production of pro-inflammatory cytokines in fat cells, causing the reduction of inflammation, while EPA is efficient in lowering triglycerides and thereby decreasing inflammation [51,52]. TNFα and IL-6 are the main cytokines affected by DHA and EPA [53]. In a clinical trial, omega-3 supplements (1250 mg/day) combined with vitamin E supplement (400 IU/day) for 12 wks. have been shown to improve fasting blood glucose and reduce insulin resistance [54,55]. Increasing the intake of omega-3 fatty acids as bioactive anti-inflammatory agents is also recommended to reduce prediabetes’s progression to full-blown type 2 diabetes [56,57]. Additionally, omega-3 fatty acids have beneficial effects on lowering cardiovascular disease risk, major causes of mortality in patients with and without diabetes [58,59]. The evidence for the protective nature of omega-3 fatty acids on the heart is abundant; present guidelines suggest that patients with CVD should consume at least 1 g of omega-3 fatty acids from either fish or fish oil supplements daily, whereas individuals without CVD are recommended to consume at least 250–500 mg daily [60].

Mental health disorders have been linked to irregularities in fatty acid structure, particularly lower concentrations of omega-3 fatty acids, and may be associated with various mental health disorders [61]. Lower omega-3 fatty acid concentrations are correlated with cerebral disorders [61]. The two main types of omega-3 fatty acids of interest are eicosapentaenoic acid (EPA) and docosahexaenoic acid (DHA) [62]. These fatty acids are vital components of cell membranes in the brain and play significant roles in neural functioning [63]. In a recent report, DHA was noted to possess a higher propensity to accumulate cholesterol-rich lipid rafts compared to EPA, in addition to having a superior potential to impact neural signaling, ultimately impacting mental health [64]. Previous reports have detected an association between depression and the low consumption of omega-3 fatty acids, while other studies show reduced levels of omega-3 fatty acids in red blood cell membranes in patients suffering from depression and anxiety [65]. The positive effects of omega-3 on mental conditions such as anxiety, depression, and the mental consequences of sleep disturbance have also been reported [56]. The key evidence for the effectiveness of the two main omega-3 fatty acids in fish oil, EPA and DHA, have been obtained in the treatment of depression and anxiety symptoms [57]. It has been shown that a daily dosage of 1.5–2 g of EPA improved depressive symptoms and enhanced mood [61]. This suggests a potential link between omega-3 fatty acid deficiencies and the development or exacerbation of these mental health conditions. While the exact mechanisms through which omega-3 fatty acids impact mental health are still being explored, it is believed that their anti-inflammatory and neuroprotective properties play a crucial role [66]. Omega-3 fatty acids are known to modulate inflammation, which is implicated in the pathophysiology of mood disorders [67]. Additionally, they influence neurotransmitter function, synaptic plasticity, and neuronal membrane fluidity, all of which are key factors in maintaining mental well-being [68].

### 3.3. Vitamin B

The vitamin B complex, including B6, B12, and folate, has numerous benefits to brain health and psychological well-being [58,69]. These water-soluble vitamins are vital for optimum brain functioning and the creation of neurotransmitters like dopamine, serotonin and GABA [59,60] while neurological disorders, including anxiety and depression, have been reported in cases of deficiency of these micronutrients [70]. It has recently been discovered that taking high doses of vitamin B6 supplements significantly reduces anxiety, stress, and depression: supplementing adults with 25 mg of vitamin B6 twice daily for six months improved symptoms of anxiety and depression [71]. Another, even more recent study reiterates the significance of vitamin B6 and supports the use of such supplements to enhance cognitive function and improve mental well-being [72]. In a longitudinal community study, individuals who had a higher dietary intake of vitamin B6 (≥1.71 mg/day for women) and B12 (≥4.79 μg/day for men) had a lower incidence of depression [73]. It has also been reported that folate and B12 deficiency can cause neurological complications, such as anxiety and depression [74]. During pregnancy, it has been found that a high level of folate, coupled with low levels of B12 is associated with gestational diabetes [75]. Conversely, B6 effectively lowers blood glucose levels among patients with gestational diabetes [76]. In addition, a study conducted by Kim et al. revealed that vitamin B6 has the potential to lower postprandial blood glucose levels after consuming sucrose and starch [77]. This effect is attributed to the inhibition of small-intestinal α-glucosidase enzyme activity. Additionally, a vitamin B complex has demonstrated the enhancement of glycemic control and renal function in patients with diabetes by reducing homocysteine levels [78].

### 3.4. Vitamin D

Vitamin D is as crucial to diabetes control and mental well-being as it is for bone health [79,80]. Studies have shown that vitamin D deficiency negatively impacts insulin sensitivity [81,82]. There is evidence that vitamin D can directly increase insulin secretions from pancreatic β-cells [83]. Additionally, supplementing with vitamin D significantly improved fasting blood glucose, insulin, and HOMA-IR in patients with diabetes [84]. Diabetes development is characterized by the ineffectiveness of insulin, modifications in pancreatic β-cells, and higher levels of inflammation [81]. Studies have identified inflammation and oxidative stress as part of the pathophysiology of type 2 diabetes and mood dysfunction [85,86,87]. When systemic inflammation occurs due to diabetes, modified functions of β-cells, including the putative stress signal to periphery, can occur due to elevated cytokines, which further stimulate insulin resistance [88]. Vitamin D lowers systematic inflammation by reducing the generation of cytokines via hindering nuclear transcription [89,90].

Emerging evidence highlights the significant role of inflammation and oxidative stress as primary contributors to the observed neuroprogression in major depressive disorder (MDD) [91]. Patients with MDD exhibit elevated inflammatory and oxidative stress biomarkers [92]. This neuroprogressive process involves stage-related neurodegeneration, cell death, reduced neurogenesis, diminished neuronal plasticity, and heightened autoimmune responses [91]. Oxidative stress results from an imbalance between reactive oxygen species (ROS) and antioxidants, disrupting intracellular redox-related signaling pathways and biomolecules. Excessive ROS lead to damage, generating pro-inflammatory molecules and triggering an immune response, ultimately causing cell death [93]. Failure to adapt to redox changes and damage from inflammatory mediators are major factors in neuroprogression and MDD [94]. Transcription factors, particularly nuclear factor (erythroid-derived 2)-like 2 and nuclear factor-κB, orchestrate the cascade of antioxidative and inflammatory events, holding relevance for MDD [95]. It has been shown that optimal levels of vitamin D cause most intracellular oxidative stress-related events to be downregulated [96]. An individual’s intracellular Nuclear factor erythroid 2-related factor 2 (Nrf2) status is associated with the buildup of mitochondrial reactive oxygen species (ROS) and an increase in oxidative stress [97,98]. In effect, Nrf2 safeguards cells from oxidative stress, which is controlled by vitamin D [99,100]. The interplay of an activated immune–inflammatory system and heightened oxidative stress in patients with diabetes supports the higher incidence of depression in this population. Vitamin D plays a pivotal role in modulating oxidative stress by influencing antioxidant defense and mitigating ROS generation. A deficiency of vitamin D among people with diabetes is linked to depression and anxiety [86]. Results of a study revealed a shared nutraceutical-gene network module between insulin resistance (IR) and depression, with central genes within this subnetwork serving as predictors for the risk of type 2 diabetes [101]. This observation underscores a potential strategy for preventing type 2 diabetes by identifying individuals at higher risk and intervening with nutraceuticals that target the core genes. For instance, regular monitoring of the vitamin D receptor (VDR) status could enable high-risk individuals to consistently assess their vitamin D levels and consider nutraceutical interventions aimed at the VDR protein as a possible preventive measure. Additionally, findings of a systematic review showed the favorable impact of vitamin D supplementation on the depression and anxiety of individuals with diabetes [102]. Thus, screening for vitamin D levels is useful in both conditions.

### 3.5. Vitamin E

The significance of vitamin E in the prevention and maintenance of diabetes and co-existing complications cannot be overemphasized. Elevated blood glucose levels contribute to heightened oxidative stress, thereby initiating and advancing the development of diabetes along with its concomitant symptoms [103]. Vitamin E is a lipid-soluble antioxidant found in cell tissues that acts as a shield against lipid peroxide, and, hence, is required for the regular functioning of immune cells [104]. Vitamin E plays a pivotal role in glucose homeostasis [105]. The vitamin improves the body’s response to insulin, which improves blood glucose levels, and acts as a potent antioxidant that safeguards cells from damage caused by oxidative stress, thereby reducing the likelihood of diabetes and its related complications [106]. In a trial of healthy adults, it was found that a high dose of vitamin E supplementation (300 mg/d) reduced lipid peroxidation [107], while another study showed that the simultaneous administration of Vitamins E and C resulted in reduced inflammation and enhanced insulin action by promoting non-oxidative glucose metabolism [108]. In several studies, when vitamin E was co-administered with atorvastatin and vitamin C in people with type 2 diabetes, blood glucose levels decreased [109,110].

The human brain is vulnerable to antioxidant deficiency, which can result in oxidative damage [111]. Deficiency of vitamin E is linked to both depression and anxiety [112], while an association between the increased intake of vitamin E (up to 15 mg/day) and a reduction in depressive symptoms has been observed [113]. The available data suggests that the optimal levels of vitamin E are contingent upon the intake of polyunsaturated fatty acids (PUFAs), including dietary linoleic acid. As a result, the precise vitamin E needs cannot be definitively determined, given that these requirements are intricately linked to the varying concentrations of PUFAs within an individual’s dietary regimen [114]. The suggested ratio between PUFA and Vitamin E, specifically alpha-tocopherol equivalents, is at least 0.6 mg alpha-tocopherol equivalents per gram of PUFA based on animal experiments [115]. This ratio is recommended to protect against the peroxidation of PUFA, particularly in relation to the unsaturated fatty acids like EPA (20:5, *n* − 3) and DHA (22:6, *n* − 3) [115].

### 3.6. Zinc

Zinc is a critical micronutrient involved in several cell functions and necessary for glycemic regulation [116]. While some studies have reported lower levels of zinc among people with diabetes [117], other reports have also emphasized that zinc deficiency is often associated with a reduced responsiveness to insulin [116]. In a recent meta-analysis (*n* = 3978 subjects), supplementation with zinc improved fasting glucose concentrations [118]. Zinc is an important cofactor in glucose homeostasis, supports the function of the immune system, and reduces oxidative stress [119]. Several complications of diabetes are linked to free radicals and raised intracellular oxidants, which are caused by decreased intracellular zinc and other antioxidants [120]. In essence, the supplementation with zinc can guard against oxidative damage at the onset of diabetes [121] and have favorable antioxidant effects, which decrease the risk of patients developing diabetes-related complications [122].

Depression and anxiety are also associated with a low intake of dietary zinc [123]. Zinc-containing neurons form a trail through the cerebral cortex, hippocampus, and amygdala, impacting mood and cognitive ability [124]. Zinc deficiency was found to be common among patients with depression and anxiety [125]. In summary, zinc is beneficial in both the prevention and management of diabetes, as well as the mental well-being of patients with diabetes.

### 3.7. Magnesium

Magnesium is a cofactor for more than 300 enzymes involved in cholesterol synthesis and glucose metabolism [126]. The association between diabetes and hypomagnesemia in extracellular and intracellular compartments is well-established [127]. Over the past decades, hypomagnesemia (serum magnesium < 0.7 mmol/L) has been strongly associated with type 2 diabetes mellitus [128]. Patients with hypomagnesemia show a more rapid disease progression and have an increased risk for diabetes complications [129]. Intracellular magnesium regulates glucokinase, KATP channels, and L-type Ca^2+^ channels in pancreatic β-cells, preceding insulin secretion [128]. Moreover, insulin receptor autophosphorylation is dependent on intracellular magnesium concentrations, making magnesium a direct factor in the development of insulin resistance [130]. Conversely, insulin is an important regulator of magnesium homeostasis [131]. Consequently, patients with diabetes and hypomagnesemia enter a vicious circle in which hypomagnesemia causes insulin resistance and insulin resistance reduces serum magnesium concentrations. It has been shown that supplementation with magnesium enhances insulin sensitivity in patients with diabetes [132]. A previous study suggested that hypomagnesemia among people with diabetes may be due to excessive urinary excretion [133]; thus, adequate dietary intake of magnesium or supplementation is recommended to maintain optimal levels [134].

Psychological disorders such as depression and anxiety also occur in individuals who have magnesium deficiency. In a randomly selected, population-based study, the relationship between magnesium and mental disorders was observed using a validated food frequency questionnaire and the General Health Questionnaire-12 to assess psychological symptoms [135]. Although there was no association between anxiety and magnesium levels, a link between a deficit of magnesium and depression was detected. Additionally, other studies noted a quicker recovery from psychiatric disorders such as insomnia [136] and bipolar disorder [137] after patients’ magnesium levels improved [138]. An intake of 125–300 mg of magnesium with meals at bedtime had also improved severe depression symptoms within a week [138,139]. Neuronal magnesium deficiency occurs when stress hormones are induced, and calcium levels are extremely high [140]. A randomized, control trial found that supplementing with magnesium aided in treating depression among people with diabetes, with no reported adverse effects [140].

### 3.8. Chromium

Chromium, an essential mineral, is important in lipid and carbohydrate metabolism and aids in improving glycemic control, and can prevent the onset of diabetes and related complications [139]. Recent studies have revealed that low levels of chromium have been associated with an increased risk of diabetes, while optimum levels of chromium enhance glucose homeostasis in patients with hyperglycemia [141]. Further, a deficiency in chromium has been linked to elevated inflammation and increased cardiometabolic risk [142]. Several RCTs have also found significant improvements in diabetes complications after serum levels of chromium were improved in the treatment groups [143,144,145]. Given the evidence, it is best to maintain optimum levels of this mineral by consuming chromium-rich foods. Good dietary sources of chromium are whole-grain foods, egg yolks, green beans, broccoli, and meats [146].

Mental health disorders have also been associated with low concentrations of chromium [147]. In a recent study, subjects aged 18–40 years randomized to receive 200 µg/d chromium experienced improvements in their anxiety and depressive conditions [148]. In a similar double-blinded study, it was found that supplementation with 400 μg/d of chromium for two weeks, followed by 600 μg/d for four weeks, reduced carbohydrate cravings among individuals with depression symptoms, while reducing mood swings [149,150].

### 3.9. Selenium

The benefits of dietary selenium in reducing the risk of diabetes are currently inconclusive [151]. While some studies have shown a positive effect in preventing the onset of diabetes [152], others have shown the opposite effect [153]. The RDA for selenium is 55 μg/d for both men and women [154]. Although some researchers claim that optimum blood concentrations of selenium are required to reduce the risk of type 2 diabetes, large cross-sectional and intervention trials confirm, rather, that supplementation among people who already have an adequate intake of selenium might increase their risk of type-2 diabetes [151,152,155]. Collectively, selenium appears to possess both beneficial and toxic effects. The preventive effect of selenium is attributed to the antioxidant role of selenoproteins and selenocysteine [156]. Conversely, excessive intake of this essential trace mineral causes selenium compounds to produce ROS [157]. In the event of oxidative stress, oxygen radicals may enhance insulin resistance and alter the function of the pancreatic β-cells [158]. The relationship between high levels of selenium and hyperglycemia is indeed complex, as individuals with high selenium levels have enhanced superoxide anion production [159,160,161]. Hydrogen peroxide, which is produced in the body, condenses to water by glutathione peroxidase; however, concerns have been raised over the lower activity of antioxidant enzymes such as glutathione peroxidase and superoxide dismutase in diabetes [162].

There is also evidence that selenium deficiency not only impacts glucose control negatively [163] but is also linked to a heightened risk of cognitive decline, depression, and anxiety [155,164,165]. In a recent study examining the relationship between reduced selenium levels and anxiety, the findings revealed a heightened risk of generalized anxiety disorder linked to lower serum concentrations of selenium [166]. Corresponding research has identified a connection between deficient selenium levels and minor and major depressive disorder [167,168]. Recent findings also suggest that selenium might have a significant role in maintaining normal neural function after experiencing stress, which is pertinent to developing and continuing anxiety disorders [169]. Therefore, it is best to have an adequate intake of this essential mineral from dietary sources.

### 3.10. Iron

A low serum iron concentration is the most common nutrient deficiency globally and increases the risk of various health conditions, including mental health disorders [170] such as anxiety, depression, and challenges with more intricate cognitive tasks [171]. The incidence of anemia among individuals with a mental disorder is significantly greater than in the general population [172]. Iron is a crucial component in the synthesis of dopamine, which is an important neurotransmitter involved in various functions such as mood regulation, motivation, and movement control [173]. Additionally, iron is essential for DNA synthesis, which is a fundamental process in the growth, repair, and maintenance of cells [174,175,176]. It has been shown that a low serum concentration of iron is associated with depression and anxiety [177]. In a recent study, it was established that a lack of iron-stimulated modifications in the hippocampus and corpus striatum, increases the risk of developing anxiety and depression [178]. Iron is a vital component of hemoglobin, as well as several enzymes in cellular metabolism that are critical for improving the central neurological structure [179]. A meta-analysis of data demonstrated a link between iron supplementation and significant enhancements in task performance, memory, and ultimately an improvement in mood and reduction of depressive symptoms [180].

In contrast, increased iron intake enhances the risk of diabetes and its complications [181]. Iron overload affects vital tissues by quickening mitochondrial decay and causing systemic free radical destruction of healthy tissues [182,183]. Excessive iron accumulation can impact critical tissues involved in glucose and lipid metabolism, such as pancreatic β cells, liver, muscle, and adipose tissue, as well as organs affected by the chronic complications of diabetes [184]. The Dietary Reference Intake (DRI) sets the Recommended Dietary Allowances (RDA) for iron to prevent anemia, ranging from 7 mg/day to 18 mg/day based on age and gender, with pregnant women needing 27 mg/day [185]. To prevent gastrointestinal issues, the Upper Level (UL) for iron is 40–45 mg/day, although toxic symptoms may arise between 20 and 60 mg/kg of iron intake [185]. Excessive iron intake has consistently been found to increase ferritin, insulin resistance, and glycosylated hemoglobin, and may also increase diabetes complications [186]. One of the distinctive functions of iron is its ability to be changeably oxidized and reduced, yet it creates a pro-oxidant molecule to produce strong reactive oxygen species [175]. In a randomized control trial, iron depletion in patients with diabetes who had high serum ferritin levels (>200 ng/mL) improved vascular dysfunction [187]. Fundamentally, maintaining iron homeostasis is critical, and an overload of iron could be a risk factor for diabetes.

**Table 1:** Recommended micronutrient doses and impacts that have been found on diabetes, depression, and anxiety.

Micronutrient	Recommended Dosage	Effects on Diabetes	Effects on Depression and Anxiety
Polyunsaturated	1250 mg/day for	Improved fasting blood	
Fatty acid [55]	for 12 wk	glucose and reduced insulin	
		resistance	
Vitamin B6 [71]	25 mg twice daily		Improved anxiety
	For six months		and depression
vitamin B12 [73]	4.79 μg/day for me		Lowered incidence of
			depression
Vitamin E [113]	15 mg/day		Reduced depression
			symptoms
Vitamin E [107]	300 mg/day	Reduces lipid peroxide thereby	
		lowering diabetes symptoms	
Magnesium [138]	125–300 mg/day		Reduced severe depression
	for one week		
Chromium [148]	200 µg/day		Improved anxiety and
			depression symptoms

## 4. Discussion

Available evidence indicates that certain nutrients may have beneficial effects on enhancing both mental health and diabetes symptoms in individuals with type 2 diabetes; however, further clinical trials are necessary to determine the optimal dosages of these nutrients for effectively addressing diabetes, as well as comorbid depression and anxiety in patients with diabetes. Additionally, it is essential to consider recommended nutrient amounts tailored to individual needs. For example, for nutrients such as vitamins B6 and B12, when administered in higher doses to research subjects with insufficient levels of the vitamins, there were significant improvements in their serum concentration levels while their symptoms also improved. On the other hand, higher doses of chromium, vitamin E, and folate, if dispensed to individuals in doses higher than the RDA, might be toxic, increasing the risk of diabetes complications and mental health disorders.

Overall, the adequate intake of the reported essential nutrients from food sources is a priority, as it has the potential to aid in glucose homeostasis, prevention or treatment of diabetes and related complications as well as mental disorders such as depression and anxiety. When dietary intake falls short, targeted supplementation with identified nutrients may offer a strategy for mitigating diabetes and mental health-related issues.

## 5. Conclusions

The evidence suggests that specific nutrients may help to concurrently optimize blood glucose control and support mental well-being. However, further research is needed to establish optimal nutrient recommendations for blood glucose control and mental health in patients with diabetes. Proper nutrition, in conjunction with appropriate medical care, can be a vital component of a comprehensive approach to both managing blood glucose levels and supporting mental health.

## Data Availability

Not applicable.
